# Spin-polarized magneto-electronic properties in buckled monolayer GaAs

**DOI:** 10.1038/s41598-018-36516-8

**Published:** 2019-02-20

**Authors:** Hsien-Ching Chung, Chih-Wei Chiu, Ming-Fa Lin

**Affiliations:** 1 Department of Physics, National Kaohsiung Normal University, Kaohsiung, 824 Taiwan; 2 Department of Physics, National Cheng Kung University, Tainan, 70101 Taiwan

## Abstract

We develop the generalized tight-binding model to fully explore the magneto-electronic properties of monolayer GaAs, where the buckled structure, multi-orbital chemical bondings, spin-orbit coupling, electric field, and magnetic field are considered simultaneously. The diverse magnetic quantization covers three groups of spin-polarized Landau levels (LLs) near the Fermi level, with the unique initial energies, LL degeneracy, energy spacings, magnetic-field-dependence, and spin splitting. Furthermore, the Landau state probabilities exhibit specific oscillation patterns, being composed of the localization centers, node regularities, and energy-dependent variations of the dominating orbitals. The density of states directly reflects the main features of the LL energy spectra in the form, height, number, and frequency of the spin-split delta-function-like prominent peaks. The electric field leads to the monotonous/nonmonotonous LL energy dispersions, LL crossing behavior, gap modulation, phase transition and enhancement of spin splitting. The complex gap modulations and even semiconductor-semimetal transitions are attributed to the strong competition among the intrinsic interactions, magnetic field, and electric field. Such predicted magneto-electronic properties could be verified by scanning tunneling spectroscopy and are helpful in designing the top-gated and phase-change electronic devices.

## Introduction

Over the past decade, graphene^1^ has successfully brought scientists into the world of two-dimensional (2D) materials based on its incredible intrinsic properties, such as high carrier mobility at room temperature (>200000 cm^2^/Vs)^2^, superior thermoconductivity (3000–5000 W/mK)^2,3^, high transparency for incident light over a wide range of wavelength (97.7%)^4^, extremely large Young’s modulus (~1 TPa) and tensile strength (~100 GPa)^5^. Few-layer graphene are observed to have diverse magnetic quantizations, e.g., the Landau levels (LLs) with the $\sqrt{{B}_{z}}$-dependent energy spectrum in monolayer graphene featuring massless Dirac fermions^6,7^, those with the linear *B*_*z*_-dependence in AB-stacked bilayer graphene featuring massive Dirac fermions^8,9^, as well as the coexistence of square-root and linear *B*_*z*_-dependent LLs in trilayer ABA stacking^10^, where *B*_*z*_ is the strength of magnetic field. Although interest in graphene materials is still high, it is also conspicuous that such systems have their limitation. For instance, in contrast to conventional semiconductors, the lack of the significant band gaps limits their potential applications for electronic devices, in which the high transistor on/off ratios are vital^11^. This obstacle urges researches to study emergent 2D materials^12^, covering group-IV^13^, group-V^14^, group III–V compounds^15^, and transition-metal dichalcogenides (TMDs)^16^. Such 2D layered materials are expected to possess various electronic properties, being sensitive to the lattice symmetry, stacking configuration, layer number, orbital hybridization, spin-orbit coupling (SOC), as well as external electric and magnetic fields.

Group-IV monoelemental 2D honeycomb materials beyond graphene, such as silicene, germanene, and stanene, have been predicted to exhibit band gaps, depending on the strength of SOC^17^. Recently, few-layer silicene, germanene, and stanene have been synthesized on distinct substrates: silicene on Ag(111)^18,19^, Ir(111)^20^, and ZrB_2_(0001)^21^; germanene on Pt(111)^22^, Al(111)^23^, and Au(111)^24^; stanene on Bi_2_Te_3_(111)^25^. Silicene, germanene, and stanene possess the buckled structures with the significant SOC’s, which grows as the atomic number increases. They are thoroughly different from the planar hexagonal graphene without SOC. Their low-lying electronic structures are dominated by the SOC’s and the multi-orbital hybridizations. In general, the group-IV materials with heavy atomic masses have the large buckling angles and rather strong SOC’s, leading to the energy gaps higher than the thermal energy at room temperature (25 meV)^17,26^. Moreover, the greatly diversified quantizations are identified from the various magnetic- and electric-field-created LLs with the non-crossing, crossing and anti-crossing behaviors^27–29^. However, the strong interactions between group-IV 2D materials and substrate might deform the buckled structure and hybridize the electronic states near the Fermi level, creating the non-negligible modifications of low-energy electronic properties. Recent experiments on tunneling spectra of silicene have evidenced the disappearance of LL sequences based on the instability from the dangling bonds of the *sp*^3^-hybridized atoms^30^.

Apart from 2D materials of group-IV elements, the binary compounds of group III-V elements have also been proposed as the honeycomb lattices with large energy gaps^15,31^. Although the group III-V elemental 2D materials of the buckled structure with the mixed *sp*^3^–*sp*^2^ bonding are more stable compared to those of planar ones with *sp*^2^ bonding^31^, the dangling-bond-induced instability remains. A promising route is to saturate the dangling bonds by halogen atoms, as successfully revealed in graphene^32^. Bulk GaAs is one of the famous group III-V elemental binary compounds, being widely used in the manufacture of electronic and optical devices due to its direct band gap (an indirect gap for silicon) and high mobility (than silicon)^33,34^. According to the first-principles calculations^35^, monolayer GaAs possesses the buckled hexagonal structure, multi-orbital chemical bonding, and significant SOC, being expected to induce the rich electronic properties and diverse magnetic quantization in the presence/absence of electric fields.

The tight-binding model proposed in ref.^35^ is extended to study the electronic properties under external electric and magnetic fields. The quantized energy spectra and wave functions are computed under the exact diagonalization method. Similar generalized tight-binding model has been widely adopted to make systematic studies on multi-dimensional carbon-based materials and hybrid systems, ranging from three-dimensional (3D) graphites^36,37^, 2D graphenes^36,38–41^, 1D graphene nanoribbons (GNRs)^42–46^, carbon nanotubes (CNTs)^47,48^, graphene nanoflake^49^ and graphene-related hybrids^50^. It is also suitable for studying the mainstream layered materials, such as group-IV^51^, group-V^52,53^, and TMD^54,55^ 2D materials.

**Figure 1:**
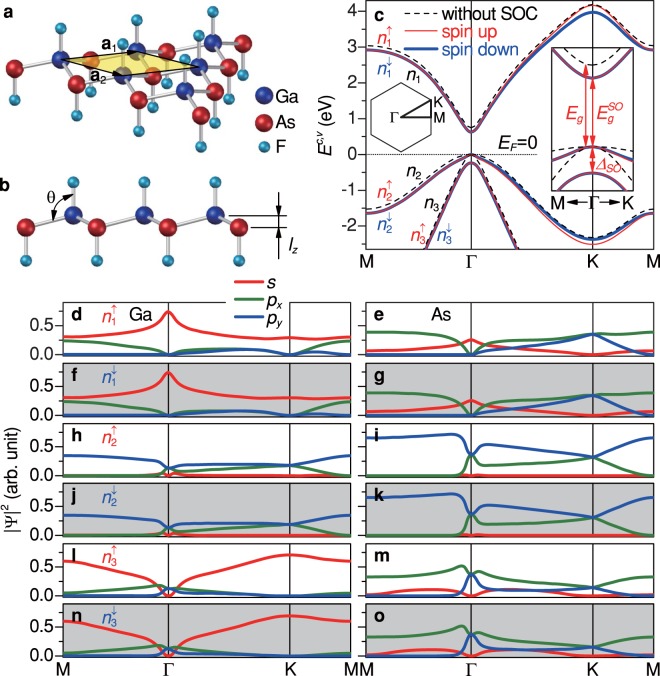
Geometric structure, low-lying subbands, and state probabilities of monolayer GaAs. (**a**) Schematic representation of the monolayer GaAs decorated by F adatoms. The unit cell is indicated by the transparent yellow rhombus, where **a**_1_ and **a**_2_ are translation vectors. The Ga, As, and F atoms are indicated by the blue, red, and cyan balls, respectively. (**b**) Side view of the low-buckled monolayer GaAs. *θ* and *l*_*z*_ are the buckling angle and the distance between the Ga-plane and As-plane, respectively. (**c**) Spin-degenerate energy subbands without SOC (*n*_1_, *n*_2_, and *n*_3_) and SOC-induced spin-polarized subbands (${n}_{1}^{\uparrow }$, ${n}_{1}^{\downarrow }$, ${n}_{2}^{\uparrow }$, ${n}_{2}^{\downarrow }$, ${n}_{3}^{\uparrow }$, and ${n}_{3}^{\downarrow }$) along the high symmetry points. The dotted line indicates the Fermi level, *E*_*F*_ = 0. (**d**–**o**) State probabilities of various orbitals located at two sublattices Ga and As with spin-up (white zones) and spin-down (gray zones) arrangements. *s*, *p*_*x*_, and *p*_*y*_ orbitals are represented by red, green, and blue curves, respectively.

In this work, the buckled monolayer GaAs with each atom being passivated by one F adatom is chosen as a model study [Fig. 1(a)], in which the saturated dangling bonds greatly weaken the substrate instability. The generalized tight-binding model, simultaneously considering geometric structure, mutli-orbital hybridizations, SOC, and external fields, is employed to explore the essential properties. The low-energy electronic properties are thoroughly investigated under the strong effects of external magnetic and electric fields. A “semimetal-semiconductor” phase diagram is obtained for the band gap as a function of both magnetic and electric fields. The current study sheds light on the diversified magnetic quantizations of the electronic energy spectra in GaAs and other group III-V 2D materials. The predicted magneto-electronic properties of the monolayer GaAs could be identified by scanning tunneling spectroscopy (STS) measurements.

## Results and Discussions

### SOC-induced spin-polarized band structure and state probabilities

Monolayer GaAs has feature-rich energy bands, mainly owing to the significant buckled structure, *sp*^3^ bonding, and SOC’s. As clearly shown in Fig. 1(c), there exist three low-lying energy subbands possessing the strong wavevector dependences in the monotonous form. The unoccupied conduction subband (*n*_1_) and two occupied valence subbands (*n*_2_ and *n*_3_), with the distinct curvatures, are initiated from the Γ point. That is to say, the low-lying electronic states could form the Γ valley being responsible for the rich magnetic quantization. Without the SOC’s, each subband is two-fold degenerate for the spin degree of freedom except that the four-fold degeneracy at the intersection of *n*_2_ and *n*_3_ subbands [dashed curves in Fig. 1(c)]. The conduction and valence subbands near the Γ point are respectively, dominated by the 4*s* and (4*p*_*x*_, 4*p*_*y*_) orbitals^35^. More importantly, a direct band gap of *E*_*g*_ = 0.742 eV is determined by the band-edge states of *n*_1_ and *n*_2_/*n*_3_ in the absence of SOC’s. The significant SOC further induces the variation of band gap and spin splitting [solid curves in Fig. 1(c)]. The band gap shrinks to ${E}_{g}^{SO}=0.623$ eV, while the *n*_2_ and *n*_3_ valence subbands are separated by Δ_*SO*_ = 0.237 eV, lifting the state degeneracy at the Γ point from four- to two-fold. The spin degeneracy is removed except for the zone from the Γ to M points. As a result, the spin-degenerate subbands become the spin-polarized ones. As for energy subbands of the same group, the splitting energies gradually grow when the electronic states deviate from the Γ point and their splittings reach the maximum values at the K (K’) points, such as 0.196 eV between ${n}_{1}^{\uparrow }$ and ${n}_{1}^{\downarrow }$ subbands, and 0.133 eV between ${n}_{2}^{\uparrow }$ and ${n}_{2}^{\downarrow }$ ones). Such spin splittings also appear in GaAs quantum wells verified by photocurrent measurements^56^, where SOC leads to interaction terms linear in wavevector **k** in the effective Hamiltonian^57^.

The electronic state probabilities (|Ψ^*c*,*v*^|^2^) correspond to the spatial distributions of different orbitals related to energy subbands and figures out the variations of major/minor orbitals along the high-symmetric points. A whole range of the orbital variations on different sublattices for the ${n}_{1}^{\uparrow \downarrow }$, ${n}_{2}^{\uparrow \downarrow }$, and ${n}_{3}^{\uparrow \downarrow }$ subbands is shown in Fig. 1(d–o). It is sufficient to discuss one of the polarized states (e.g., spin-up states), since the state configurations of spin up (white zones) and spin down (gray zones) states are quite similar. The state probabilities for different orbitals are very sensitive to the sublattices and wavevectors. In the conduction ${n}_{1}^{\uparrow \downarrow }$, valence ${n}_{2}^{\uparrow \downarrow }$, valence ${n}_{3}^{\uparrow \downarrow }$ subbands, the *s*-orbitals (red curves), *p*_*y*_-orbitals (blue curves), and *p*_*x*_-orbitals (green curves), respectively, make the most important contributions for a wide range of wavevectors. Remarkably, the *p*_*x*_- and *p*_*y*_-orbitals are of identical intensity at the high-symmetric Γ and K points. The state probabilities near the Γ point, which are much different from those far away it, obviously reveal the dramatic orbital variation for the low-lying states. The conduction subbands are dominated by the *s*-orbitals, whose state probabilities on the Ga sublattice is larger than those on the As sublattice [Fig. 1(d–g)]. The increase of *p*_*x*_- and *p*_*y*_-orbital strength and the decrease of *s*-orbital strength come to exist as **k** deviates from the Γ point. The valence *n*_2_ (*n*_3_) subbands are dominated by *p*_*y*_-orbitals (*p*_*x*_-orbitals) [Fig. 1(h–o)]. Instead of the Ga sublattices, the dominating orbitals on the As sublattices possess the larger strength. It should be noted that the relative strength of the orbital probabilities for the low-lying states will reflect on the quantized magneto-electronic states. In other words, the low-energy Landau states features those accumulated zero-field states near the Γ valley (discussed later).

### Spin-polarized LL spectra and state probabilities

**Figure 2:**
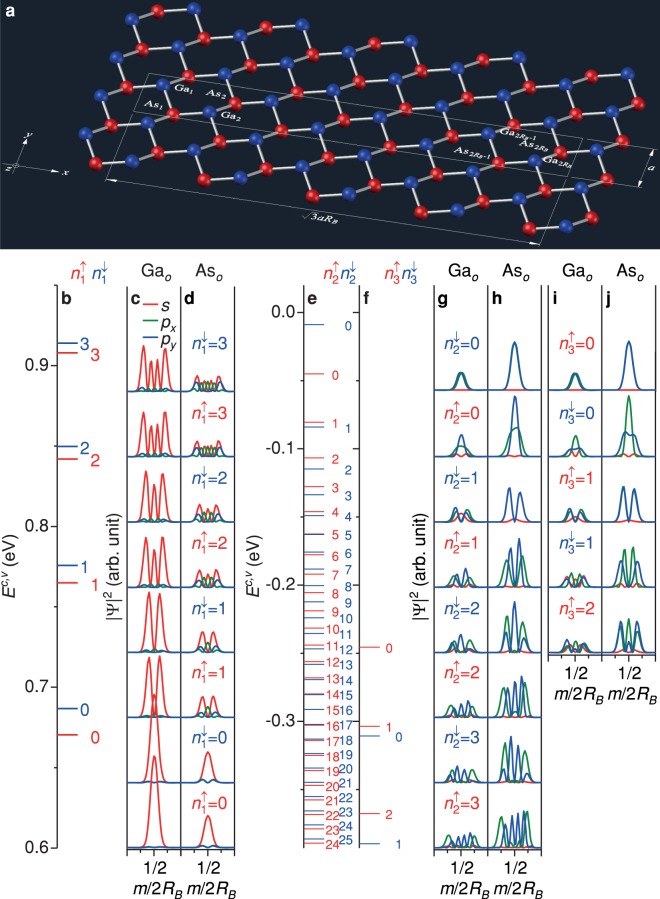
Spin-polarized LLs and state probabilities. (**a**) Geometric structure of the low-buckled monolayer GaAs in a uniform perpendicular magnetic field of ${B}_{z}\hat{z}$. The enlarged unit cell, with 2*R*_*B*_ Ga and 2*R*_*B*_ As atoms, satisfies the periodicity of Peierls phase, where *R*_*B*_ is the ratio of flux quantum to magnetic flux through a hexagon. The F adatoms are omitted for a clear presentation. (**b**,**e**,**f**) Three groups of spin-polarized dispersionless LLs are shown under *B*_*z*_ = 60 T. One group of unoccupied conduction LLs (${n}_{1}^{\uparrow }$ and ${n}_{1}^{\downarrow }$) and two groups of occupied valence LLs (${n}_{2}^{\uparrow }$, ${n}_{2}^{\downarrow }$, ${n}_{3}^{\uparrow }$, and ${n}_{3}^{\downarrow }$). (**c**,**d**,**g**–**j**) The corresponding probabilities of the subenvelope functions near the localization center. *s*, *p*_*x*_, and *p*_*y*_ orbitals are indicated by red, green, and blue curves, respectively. The major properties, such as the dominating orbitals, node regularities and localization centers, are the same for various strengths of magnetic field, indicating that the main features of state probabilities can be discussed only for a specific *B*_*z*_.

Magnetic fields constrain carrier motions in the real space and lead to the quantization of the cyclotron orbitals. They create the highly-degenerate dispersionless states, being called the Landau levels (LLs). The LL initial energies might be related to the zero-field electronic structures. The energy spacing between Landau states depends on the external fields, massless/massive characteristic of the Dirac fermions, and SOC’s. Near the Fermi energy, there are three groups of spin-polarized dispersionless LLs, i.e., one group of unoccupied conduction LLs [${n}_{1}^{\uparrow }$ and ${n}_{1}^{\downarrow }$ in Fig. 2(b)] and two groups of occupied valence LLs [${n}_{2}^{\uparrow }$, ${n}_{2}^{\downarrow }$, ${n}_{3}^{\uparrow }$, and ${n}_{3}^{\downarrow }$ in Fig. 2(e,f)]. The distinct spin polarization in each group of LLs results from the SOC’s between the 4*p*_*x*_ and 4*p*_*y*_ orbitals. Such groups are, respectively, initiated near 0.62 eV, 0 eV, and −0.24 eV, which reflect the electronic state energies at the Γ point in the absence of magnetic fields. For each (*k*_*x*_, *k*_*y*_), all LLs are two-fold degenerate. For each (*k*_*x*_, *k*_*y*_), all LLs are two-fold degenerate, being attributed to the one Γ-valley degree of freedom and two *B*_*z*_-field-direction degree of freedom. As the state energy grows, the energy spacing between LLs of the same spin-up/spin-down subgroup gradually shrinks.

Wave functions, presenting the spatial information of electronic states, are very important in realizing fundamental physical properties, such as charge densities^58–61^, state mixing^62,63^, and optical selection rules^45,46,64,65^. Under the influence of magnetic fields, wave functions in monolayer GaAs exhibit the peculiar spatial distributions, where the localization center, the dominating orbital, the oscillational form, the number of zero points are very sensitive to the wavevector, state energy, and spin configurations. Each spin-polarized LL wave function can be decomposed into subenvelope functions with the (*s*, *p*_*x*_, *p*_*y*_) orbitals on the Ga and As sublattices at the odd and even sites. For the sake of simplicity, only the distribution probabilities of subenvelope functions at the odd sites (Ga_*o*_ and As_*o*_) will be considered because the even-site probabilities have the same behavior as the odd-site ones. The localization centers of the LL wave functions are strongly dependent on the wavevectors. At (*k*_*x*_, *k*_*y*_) = (0, 0), the doubly degenerate spin-polarized LL states are localized at the 1/2 and 0 positions of the enlarged unit cell (*m*/2*R*_*B*_ = 1/2 and 0). The former is chosen for a model study, since both of them have the state probabilities.

The probabilities of subenvelope functions are well-behaved in their spatial distributions. Their oscillation patterns around the localization center are similar to those of harmonic oscillators, clearly revealing regular node (zero-point) numbers. For any particular LL, the node number of the *s*-/*p*_*x*_-/*p*_*y*_-decomposed orbital subenvelope function is identical for the Ga and As sublattices. The subenvelope function of the dominating orbital is significant for characterizing the Landau state, and its node number, which gradually grows as the state energy increases, could serve as the quantum number of the LL. In the *n*-th conduction LLs (${n}_{1}^{\uparrow }=n$ and ${n}_{1}^{\downarrow }=n$), the state probabilities of dominating *s*-orbital subenvelope functions, with *n* zero points, are much stronger than those of *p*_*x*_ and *p*_*y*_-decomposed ones, with *n* + 1 nodes [Fig. 2(c,d)]. In the ${n}_{2}^{\uparrow }=n$ (${n}_{2}^{\downarrow }=n$) valence LLs, there are *n*, *n*, and *n* + 1 (*n* − 1) nodes in the major *p*_*y*_-orbitals and minor *p*_*x*_- and *s*-orbitals, respectively [Fig. 2(g,h)]. In the ${n}_{3}^{\uparrow }=n$ (${n}_{3}^{\downarrow }=n$) valence LLs, *n*, *n*, and *n* − 1 (*n* + 1) nodes, respectively, correspond to the dominant *p*_*x*_-orbitals and minor *p*_*y*_- and *s*-orbitals [Fig. 2(i,j)].

It is noteworthy that the Landau state reflects the average of the accumulated neighboring zero-field electronic states with very close energies. In other words, the relative strength among LL subenvelope function probabilities of distinct orbitals and sublattices directly arise from that of the zero-field wave functions. In each LL group, the energy-dependent relative orbital strength is closely related to the **k**-dependent one at *B*_*z*_ = 0, mainly owing to the monotonous band structure near the Γ point. The dominant *s*-orbitals on the Ga sublattice have strength stronger than those on the As sublattice in the conduction LLs. Furthermore, the enhancement of *p*_*x*_- and *p*_*y*_-orbital strengths, accompanied with the decline of *s*-orbital strength, take place as ${n}_{1}^{\uparrow }$ and ${n}_{1}^{\downarrow }$ grow [comparison between Figs 2(c,d) and 1(d–g)]. Instead of the Ga sublattice, the dominating orbitals on the As sublattice in the valence LLs exhibit the higher strengths. The *p*_*x*_- and *p*_*y*_-orbitals on a specific sublattice are of the same strength in the ${n}_{2}^{\downarrow }=0$ and ${n}_{3}^{\uparrow }=0$ valence LLs [Fig. 2(g–j)], reflecting the fact that the zero-field *p*_*x*_- and *p*_*y*_-orbitals possess the equivalent strength at the Γ point [Fig. 1(h–o)]. As the subband index increases, the ${n}_{2}^{\uparrow \downarrow }$ (${n}_{3}^{\uparrow \downarrow }$) valence LLs become *p*_*y*_-orbital- (*p*_*x*_-orbital-) dominated, which resembles the **k**-dependance of dominated orbitals near the Γ point. The aforementioned LL node regularities and energy-dependent orbital variation provide the fundamental information for further researches in optical and transport properties, such as magneto-optical absorption selection rules including major/minor optical transitions and the available/forbidden transport channels.

### Magnetic field dependence of LL spectra and DOS

**Figure 3:**
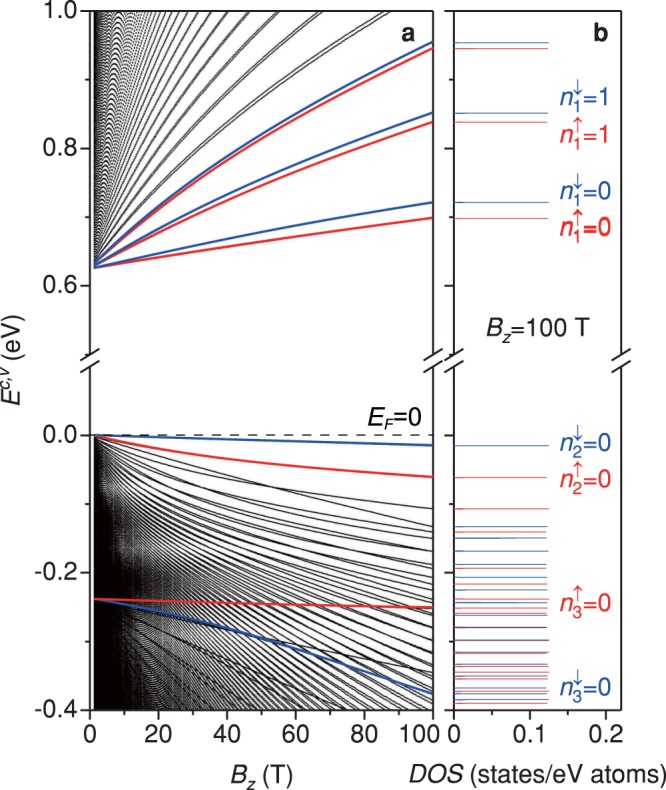
Magnetic-field-dependent LLs and DOS. (**a**) The LL energies have no simple dependence on the strength of magnetic field. With the increment of *B*_*z*_, the spin splitting between the ${n}_{i}^{\uparrow }=n$ and ${n}_{i}^{\downarrow }=n$ LLs is enhanced, and the gap between the lowest conduction ${n}_{1}^{\uparrow }=0$ LL and the highest valence ${n}_{2}^{\downarrow }=0$ LL grows. Part of the spin-up (red lines) and spin-down (blue lines) LLs are indicated for guidance. The Fermi level is marked by the dash line. (**b**) Spin-polarized DOS at *B*_*z*_ = 100 T. The delta-function-like peaks correspond to the dispersionless and highly-degenerate features. Those due to the spin-up and spin-down states are depicted in red and blue, respectively.

The low-lying LL energies exhibit a monotonic variation with the strength of magnetic field, directly reflecting the feature of the monotonic band structure near the Γ point at zero field [Fig. 3(a)]. A simple relation between the LL energies and *B*_*z*_ is absent; that is, there are no linear or square-root *B*_*z*_-dependences, as revealed in graphene systems. Between the *n*-th spin-up and spin-down LLs of the same group (${n}_{i}^{\uparrow }={n}_{i}^{\downarrow }=n$; $i\in \{1,2,3\}$), their energy spacing grows in the increment of *B*_*z*_, arising from the enhanced SOC by the more localized LL wave functions. For instance, the energy spacing is 24 meV between ${n}_{1}^{\uparrow }=0$ and ${n}_{1}^{\downarrow }=0$ LLs at *B*_*z*_ = 100 T (comparable to the thermal energy at room temperature). Under a very small magnetic field (*B*_*z*_ → 0), the energy spacing between the lowest conduction LL and the highest valence LL approaches (or converges) to the zero-field energy gap. For an increasing magnetic field, these two LLs deviate from the Fermi level, thus leading to the increase of energy gap. Most importantly, the non-crossing and crossing behaviors appear in the *B*_*z*_-dependent energy spectra. The first spin-up and spin-down groups always have the lower and higher energies, respectively, so that these two subgroups do not cross each other during the variation of *B*_*z*_. However, the distinct spin LLs might exhibit the crossing phenomena, especially for the second and third groups. The significant features of LL energy spectra further illustrate the strong competitions/cooperations among the lattice symmetry, multi-orbital chemical bondings, SOC’s, and magnetic field.

The DOS, defined as^45,46,66^ ${\sum }_{k}\,{\sum }_{{n}_{i}^{\uparrow \downarrow };i\in \{1,2,3\}}\,\delta [\omega -{E}^{c,v}({\bf{k}},{n}_{i}^{\uparrow \downarrow })]$, directly reflects the main features of the LL energy spectra as depicted in Fig. 3(b). The dispersionless and highly-degenerate LLs can create the delta-function-like prominent peaks as the van Hove singularities in DOS. Three groups of delta-function-like symmetric peaks, respectively, appear from ~0.62 eV, 0 eV, and −0.24 eV. Their peak heights are the same, indicating the identical degeneracy of LLs. In each spin-polarized LL subgroup, the peak spacing is shrunk for a larger subband index. The above-mentioned characteristics of LL peaks, including peak structure, height, and spacing, could be verified through the experimental measurements using STS^6,7^. Moreover, it is expected that the optical absorption peaks are contributed by the specific inter-LL transitions with the regularly spatial probability distributions.

### Electric field dependence of LL spectra

**Figure 4:**
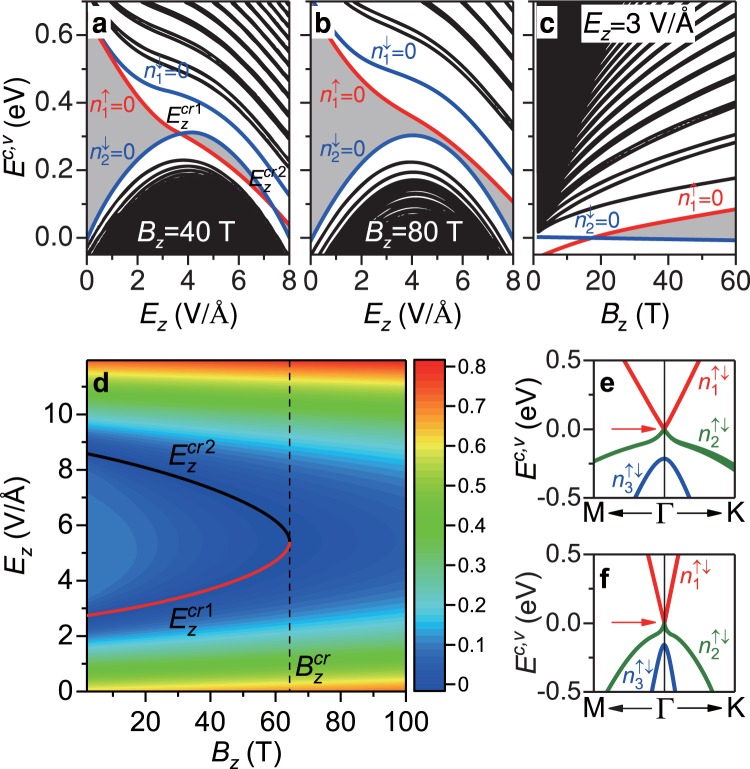
External-fields-dependent LLs, gap modulation and phase transition. (**a**,**b**) Electric-field-dependent LL energy spectra at *B*_*z*_ = 40 and 80 T. The monotonous/nonmonotonous dispersion relations, intergroup LL crossing, enhancement of spin splitting, and gap modulation are obviously shown. (**c**) Magnetic-field-dependent LL energies at *E*_*z*_ = 3 V/Å. (**d**) The *E*_*z*_-*B*_*z*_ color contour diagram clearly illustrates the complex gap modulation and phase transition. The red and black curves, respectively, indicate ${E}_{z}^{cr1}$ and ${E}_{z}^{cr2}$ at a specific magnetic field *B*_*z*_. (**e**,**f**) In the absence of magnetic field, the zero gap takes place at *E*_*z*_ = 2.59 V/Å and *E*_*z*_ = 8.75 V/Å (arrows), indicating the strength of the critical electric fields ${E}_{0}^{cr1}$ and ${E}_{0}^{cr2}$ at *B*_*z*_ → 0. The ${n}_{1}^{\uparrow \downarrow }$, ${n}_{2}^{\uparrow \downarrow }$, and ${n}_{3}^{\uparrow \downarrow }$ subbands are colored in red, green, and blue, respectively.

The magneto-electronic properties of monolayer GaAs with buckled structure, being thoroughly different from those of monolayer graphene with planer structure, can be diversified by a perpendicular electric field, *E*_*z*_. The Coulomb potential difference *V*_*z*_ = *E*_*z*_*l*_*z*_ between the planes of Ga and As sublattices clearly lead to the monotonous/nonmonotonous dispersion relations, crossing LL spectra, enhancement of spin splitting, and modulation of energy gap. For a moderate magnetic field [Fig. 4(a)], the ${n}_{1}^{\uparrow \downarrow }$ and ${n}_{2}^{\uparrow \downarrow }$/${n}_{3}^{\uparrow \downarrow }$ LL energy spectra, respectively, exhibit monotonous decrease and increase as the electric field grows. An intergroup LL crossing takes place between ${n}_{1}^{\uparrow }=0$ and ${n}_{2}^{\downarrow }=0$ LLs at the critical electric fields (${E}_{z}^{cr1}=3.6$ V/Å and ${E}_{z}^{cr2}=7.3$ V/Å), where the variation of energy gap is indicated by the gray zone. The former and the latter, respectively, become the occupied and unoccupied states after ${E}_{z}^{cr1}$, and return to their original states after ${E}_{z}^{cr2}$, so that monolayer GaAs exhibits the gapless behavior at the specific critical fields of ${E}_{z}^{cr1}$ and ${E}_{z}^{cr2}$. Meanwhile, the spin splitting is enhanced; furthermore, the energy spacing between the ${n}_{1}^{\uparrow }=0$ and ${n}_{1}^{\downarrow }=0$ LLs is about 100 meV (much higher than the room-temperature thermal energy). For a large magnetic field [Fig. 4(b)], the ${n}_{1}^{\uparrow \downarrow }$ LLs present a monotonous decline, while the ${n}_{2}^{\uparrow \downarrow }$/${n}_{3}^{\uparrow \downarrow }$ LLs vary nonmonotonously with the turning points at *E*_*z*_ ~ 4 V/Å. The energy gap gradually shrinks and then reaches a minimum finite value (*E*_*g*_ = 26 meV) without the intergroup crossing between ${n}_{1}^{\uparrow \downarrow }$ and ${n}_{2}^{\uparrow \downarrow }$ LLs. There is no semiconductor-metal transition during the variation of *E*_*z*_ under a very high *B*_*z*_. Moreover, the *B*_*z*_-dependent LL energy spectrum clearly shows the similar intergroup crossing at the critical magnetic field under a sufficiently high electric field, e.g., $({B}_{z},{E}_{z}^{cr1})=(18\,{\rm{T}},3{\rm{V}}/\backslash {\rm{AA}})$ in Fig. 4(c). In short, the electric and magnetic fields induce complex variations of the energy gap; therefore, their strong competitions create the semiconductor-semimetal transitions only under the critical fields.

The gap modulation owing to the competition between magnetic and electric fields is presented in detail by the color map as shown in Fig. 4(d). For a magnetic field lower than the critical one (${B}_{z} < {B}_{z}^{cr}\sim 64$ T), the gap gradually shrinks to zero, becomes finite, reduces to zero again, and then grows as *E*_*z*_ increases. In this variation, the intergroup LL crossings between ${n}_{1}^{\uparrow }=0$ and ${n}_{2}^{\downarrow }=0$ LLs take place at ${E}_{z}^{cr1}$ and ${E}_{z}^{cr2}$, which stands for the occurrence of the semiconductor-semimetal transitions at these critical electric fields. For ${B}_{z} > {B}_{z}^{cr}$, the gap gradually reduces to a finite size and than increases as *E*_*z*_ grows. In addition, ${E}_{0}^{cr1}\sim 2.59$ V/Å and ${E}_{0}^{cr2}\sim 8.75$ V/Å at *B*_*z*_ → 0 can be straightforwardly figured out by the energy spectrum at *B*_*z*_ = 0, owing to the direct correspondence between the LL initial energies at small *B*_*z*_ and the energies of electronic states at the Γ point at *B*_*z*_ = 0 [Fig. 4(e,f)]. The aforementioned external-field-controlled gap modulation and phase transitions are helpful in developing the top-gated electronic/optical devices and enable potential applications in phase-change electronic devices^67^.

### The experimental verifications of the theoretical predictions

The main characteristics and the external field-induced modulations of the prominent symmetric Landau peaks in the DOS could be verified by the experimental measurements of STS. This experiment is an extension of scanning tunneling microscopy (STM)^68^ and provides the detailed informations about the DOS’s on a sample surface, such as silicon^69^ and CNTs^70^. The tunneling differential conductance (*dI*/*dV*), being roughly proportional to the DOS^71^, directly reveals the van Hove singularities of the electronic energy spectra, such as, the form, number, position and intensities of the special structures. Part of theoretical predictions on the LL energy spectra of few-layered graphene have been identified from the STS measurements, e.g., the $\sqrt{{B}_{z}}$-dependent LL energies in monolayer graphene^6,7^, the linear *B*_*z*_-dependent LL spectra in AB-stacked bilayer graphene^8,9^, and the concurrence of square-root and linear *B*_*z*_-dependences in trilayer ABA stacking^10^. The predicted magneto-electronic properties of the monolayer GaAs, including three groups of LLs without a simple *B*_*z*_-dependence, the external-field-controlled gap modulation and the SOC-induced spin splitting, could be directly examined from the STS experiments. Such verifications are very useful in fully understanding the very strong competitions/cooperations among the critical factors.

The aforementioned main features of wave functions could be confirmed by the spectroscopic-imaging STM^72^, which is available in resolving the spatial charge distributions from the local DOS. This appropriate experimental technique is very suitable for identifying standing waves and Landau wave functions on the surfaces of various condensed-matter systems. Standing waves have been directly observed at the surface steps of Au(111) and Cu(111)^73^, as well as finite-length metallic CNT^74^. Also, the spatial mapping of the electronic states in the troughs between self-organized Pt nanowires on Ge(001) is presented^75^. Recently, the Landau orbits without nodes have been observed^76^, and subsequently, observations of the concentric-ring-like nodal structures are also obtained^77^. In monolayer GaAs, the predicted orbital domination for various groups of LLs and the relative strength of various orbitals (or different sublattices) for a specific LL could be examined through spectroscopic-imaging STM measurements on nodal structures.

### Comparisons among the buckled GaAs, planar graphene, and other buckled group-V honeycomb lattices

Monolayer graphene and GaAs possess very different electronic properties and responses to external fields. For instance, the low-energy electronic structure of the former exhibits a pair of single-orbital-dominated (*p*_*z*_) conduction and valence bands, in which they linearly intersect at the K/K’ points and are doubly degenerate in the spin degree of freedom. This zero-gap system sharply contrasts the middle-gap monolayer GaAs. The latter exhibit a direct gap of 0.623 eV at the Γ point, being related to the multi-orbital-induced parabolic bands (*s*, *p*_*x*_, *p*_*y*_ orbitals). Moreover, the significant SOC’s and *sp*^3^ bondings create the **k**-dependent spin-polarized energy subbands. The distinct electronic properties are also revealed in rich magnetic quantizations, such as the magnetic field dependence of LL energies, spin splitting/non-splitting, localization centers of Landau wave functions, non-crossing and crossing behaviors, and quantum mode regularities (i.e., number and ordering of node in the LL wave functions), and the electric-field effects. The LLs directly reflect the main features of zero-field energy dispersions, revealing $\sqrt{{B}_{z}}$-dependent spin-degenerate LLs and the nonspecific-*B*_*z*_-dependent spin-split LLs in monolayer graphene and GaAs, respectively. The localization centers of the spin-degenerate states (spin-polarized states) are situated at 1/6, 2/6, 4/6, and 5/6 (0 and 1/2) positions of the enlarged unit cell. Only the valence LLs of GaAs display the crossing phenomena during the variation of *B*_*z*_. For a specific LL of graphene (GaAs), the major node numbers of subenvelope functions in different sublattices differ by one (are identical). Electric fields further induce an on-site energy difference between two buckled sublattices of GaAs, leading to the gap modulation, phase transition, and enhancement of spin splitting.

According to the theoretical predictions, monolayer group-V systems, such as bismuthene^78^ and phosphorene^53^, exhibit the rich and unique magneto-electronic properties. The former is similar to monolayer GaAs in the presence of SOC’s. However, the strength of SOC in Bi system (~1.5 eV) is much higher than that (~0.1 eV) of GaAs. The spin-up- and spin-down-dominated LL energy spectra are predicted to be split very obviously. Its low-lying electronic structures covers one conduction band and two valence bands, arising from the (*p*_*x*_, *p*_*y*_, *p*_*z*_) orbitals. Specifically, the *p*_*z*_ orbital, but not the *s* one, also plays an important role in the essential electronic properties. The first valence band nearest to *E*_*F*_ belongs to the oscillatory dispersion, and the other two have the parabolic dispersions. There exists an indirect energy gap of ~0.293 eV^78^. The multi-constant-energy loops could lead to the abnormal *B*_*z*_-dependent energy spectrum with the frequently anti-crossing and crossing phenomena, and the non-well-behaved LL wave functions with the major and minor modes (the perturbed LLs). An electric field in the heavily buckled bismuthene is expected to induce more perturbed LLs and thus the frequent anti-crossing behaviors for any spin-split subgroups.

## Concluding Remarks

We develop the generalized tight-binding model from ref.^35^ to explore the magneto-electronic properties of monolayer GaAs. This theoretical framework could be further utilized to investigate the diverse magnetic quantizations of the emergent 2D layered materials. Plenty of critical factors, including the buckled structure, multi-orbital hybridizations, SOC’s, electric field, and magnetic field, are considered in the calculations simultaneously. Three groups of SOC-induced spin-polarized subbands (${n}_{1}^{\uparrow \downarrow }$, ${n}_{2}^{\uparrow \downarrow }$, ${n}_{3}^{\uparrow \downarrow }$) initiated from the Γ point exhibit monotonous energy dispersions and strong **k**-dependent spin splitting. There are a direct band gap (${E}_{g}^{SO}=0.623$ eV) between ${n}_{1}^{\uparrow \downarrow }$ and ${n}_{2}^{\uparrow \downarrow }$ subbands as well as a SOC-induced energy splitting (Δ_*SO*_ = 0.237 eV) between ${n}_{2}^{\uparrow \downarrow }$ and ${n}_{3}^{\uparrow \downarrow }$ subbands at the Γ point. The electronic state probabilities demonstrates that the conduction ${n}_{1}^{\uparrow \downarrow }$, valence ${n}_{2}^{\uparrow \downarrow }$, valence ${n}_{3}^{\uparrow \downarrow }$ energy subbands are *s*-, *p*_*y*_-, *p*_*x*_-orbital-dominated, respectively. The calculate results clearly show that monolayer GaAs is in great contrast with group-IV and group-V layered systems in the essential magneto-electronic properties, covering the localization centers, state degeneracy, orbital-dependent subenvelope functions, spin splittings, *B*_*z*_- and *E*_*z*_-dependences, and non-crossing/crossing/anti-crossing phenomena. These important differences are attributed to the distinct multi-orbital hybridizations and SOC’s.

Magnetic quantization induces three groups of spin-polarized LLs with initial energies respectively near 0.62 eV, 0 eV, and 0.24 eV, reflecting the zero-field electronic state energies at the initial Γ point. Each LL is doubly degenerate based on one Γ-valley and two *z*-axis degree of freedom. The magnetic state probabilities are well-behaved in their spatial distributions, possessing oscillation patterns with regular nodes at the **k**-dependent localization centers (e.g., the 0 and 1/2 positions of the enlarged unit cell for (*k*_*x*_, *k*_*y*_) = (0, 0)), as revealed by a harmonic oscillator. In each LL, the node numbers of various orbital subenvelope functions on the Ga and As sublattices are identical, and the *s*- and (*p*_*x*_, *p*_*y*_)-orbital node numbers differ from each other by one. Such predicted characteristics of magnetic wave functions could be examined through spectroscopic-imaging STM measurements on nodal structures. Moreover, the non-specific *B*_*z*_-dependence of the LL energy spectrum obviously illustrates the significant multi-orbital bondings and SOC’s. Both energy gap and energy spacing of spin splitting are gradually enhanced in the increase of magnetic field. The conduction LLs only exhibit the non-crossing behavior during the variation of *B*_*z*_. However, the inter-subgroup LL crossings happen in the first/the second valence group, and they frequently occur between these two valence groups. There are six subgroups of spin-polarized LL DOS peaks featuring the rich van Hove singularities. The delta-function-like symmetric structure, the initial frequencies for each subgroup, the degeneracy-induced peak height, and the reduced energy spacing of the higher quantum numbers, could be examined from the STS measurements.

The electric field causes the drastic changes in energy dispersions, LL crossings, enhancement of spin splitting, gap modulation, and phase transition. For a magnetic field lower than the critical one, ${B}_{z} < {B}_{z}^{cr}$, the intergroup LL crossings occur between the conduction and valence LLs at the critical electric fields, inducing semiconductor-semimetal transitions. On the other hand, energy remains finite in the absence of the intergroup LL crossing near the Fermi level. It should be noted that the spin splitting is enhanced with an energy spacing larger than the room-temperature thermal energy. The complex gap modulations and phase transitions based on the rather strong competitions/cooperations between magnetic and electric fields are investigated in detail. The *E*_*z*_-*B*_*z*_ phase diagram clearly illustrate the critical electric fields under a specific magnetic field. Specifically, the *E*_*z*_-induced LL anti-crossings are absent, being thoroughly different those in few-layer group-IV and group-V systems.

## Method

### Geometric structure

Monolayer GaAs has a buckled honeycomb lattice with each atom being passivated by an F adatom, as clearly shown in Fig. 1(a). There exists the *sp*^3^ chemical bonding among (Ga, As, F) atoms (three for As or Ga; one for F), in which the Ga-As bond length is about 2.521 Å. A unit cell containing two different Ga and As sublattices is indicated by the rhombus with the primitive unit vectors, **a**_1_ and **a**_2_ of a lattice constant *a* = 4.226 Å. The altitude of the buckled structure measured from the distance between the Ga- and As-plane is *l*_*z*_ = 0.633 Å [Fig. 1(b)]. The buckling angle *θ* between the Ga-As bond and the *z*-axis is about 104.54°. This configuration is free from dangling bonds and thus chemically stable. F atoms are right above/below the Ga/As atoms with the Ga-F and As-F distances of 1.776 Å and 1.781 Å, respectively^35^.

### Generalized tight-binding model

The generalized tight-binding model, being very suitable for exploring the essential properties of monolayer GaAs, is developed, where many critical factors, the buckled structure, multi-orbital hybridizations, SOC, electric field, and magnetic field, are included in the calculation simultaneously. The current model provides a theoretical framework for investigating the strong competitions/cooperations among various critical factors and affords systematic studies from multi-dimensional materials to hybrid systems. The theoretical models, with both single-particle and many-body schemes, can also be combined to comprehend the essential physical properties, e.g., frequency-dependent and static Kubo formulas for exploring the optical absorption spectra^40,45,46,64^ and quantum Hall effect, respectively.

To illustrate the electronic properties explicitly, the Hamiltonian built from the tight-binding functions of 4 *s*, 4*p*_*x*_, and 4*p*_*y*_ orbitals is expressed as

1 $$ {\mathcal H} =\sum _{m,\alpha }\,{\varepsilon }_{m}^{\alpha }{c}_{m}^{\alpha \dagger }{c}_{m}^{\alpha }+\sum _{\langle m,n\rangle ,\alpha ,\beta }\,{\gamma }_{mn}^{\alpha \beta }({c}_{m}^{\alpha \dagger }{c}_{m}^{\beta }+h.\,c.\,\,),$$

where ${\varepsilon }_{m}^{\alpha }$, ${c}_{m}^{\alpha \dagger }$, and ${c}_{m}^{\alpha }$ respectively represent the on-site energy, creation, and annihilation operators of an electron at the *α*-orbital of the *m*-th atom. ${\gamma }_{mn}^{\alpha \beta }$ is the nearest-neighbor hopping integral between an *α*-orbital of the *m*-th atom and a *β*-orbital of the *n*-th atom. The multi-orbital hopping integrals are ${\gamma }_{mn}^{ss}={V}_{ss\sigma }$, ${\gamma }_{mn}^{s{p}_{x}}={V}_{sp\sigma }\,\cos \,{\theta }_{x}$, ${\gamma }_{mn}^{s{p}_{y}}={V}_{sp\sigma }\,\cos \,{\theta }_{y}$, ${\gamma }_{mn}^{{p}_{x}{p}_{x}}={V}_{pp\sigma }\,{\cos }^{2}\,{\theta }_{x}+{V}_{pp\pi }(1-{\cos }^{2}\,{\theta }_{x})$, ${\gamma }_{mn}^{{p}_{y}{p}_{y}}={V}_{pp\sigma }\,{\cos }^{2}\,{\theta }_{y}+{V}_{pp\pi }(1-{\cos }^{2}\,{\theta }_{y})$, and ${\gamma }_{mn}^{{p}_{x}{p}_{y}}=({V}_{pp\sigma }-{V}_{pp\pi })\,\cos \,{\theta }_{x}\,\cos \,{\theta }_{y}$, where *θ*_*x*_ and *θ*_*y*_ are respectively the angles of the vector initiated from the *m*-th atom to the *n*-th atom with respect to the *x*- and *y*-axis^79^, and the Slater-Koster hopping parameters in the *sp*^3^ bonding optimized at the equilibrium state are *V*_*ssσ*_ = −1.707 eV, *V*_*spσ*_ = 2.056 eV, *V*_*ppσ*_ = 2.650 eV, and *V*_*ppπ*_ = −0.827 eV^35^. The on-site energies of *s*- and *p*-orbitals are set to the values (−12.00 eV, −5.67 eV) for Ga and (−17.68 eV, −8.30 eV) for As, being taken from those of bulk GaAs^80^. It should be noted that the 4*p*_*z*_ orbitals are ignored in the Hamiltonian in Eq. 1. A strong fluorination could fully suppress the dangling bands of monolayer GaAs; that is, there exist very pronounced between 2*p*_*z*_ and 4*p*_*z*_ orbitals due to the top-site adsorptions. The rather stable 2*p*_*z*_ − 4*p*_*z*_ chemical bonds create the deeper valence bands far away from the Fermi level^81,82^. This is responsible for the absence of 2*p*_*z*_ and 4*p*_*z*_ orbitals in the current model. The *σ* orbitals (*p*_*x*_, *p*_*y*_) with the stronger SOC dominate low-lying electronic states, instead of *π* orbitals (*p*_*z*_) with weaker SOC. The similar phenomena are also revealed in the fluorinated graphene theoretically^83–85^ and experimentally^32^, in which the (2*p*_*x*_, 2*p*_*y*_) orbitals of carbon atoms make important contributions to the low-lying band structures, but not 2*p*_*z*_ orbitals. As a result, the *π* bonding-related linear Dirac-cone structures are suppressed thoroughly.

### Spin-orbit coupling

When an electron of momentum **p** moves close to the atomic nuclei in a crystal with an electric potential *V*, it experiences an effective magnetic field ${B}_{{\rm{eff}}}\sim \nabla V\times {\bf{p}}/{m}_{0}{c}^{2}$ in its rest-frame, where *m*_0_ is the mass of a free electron and *c* is the speed of light. Such field induces a momentum-dependent Zeeman energy called the SO coupling, being expressed

2 $${H}^{SO}=\frac{\hslash }{4{m}_{0}^{2}{c}^{2}}(\nabla V\times {\bf{p}})\cdot \sigma ,$$

where $\hslash$ is the reduced Planck constant and *σ* is the vector of Pauli matrices. In the central field approximation, the crystal potential *V*(**r**) is considered as the spherical atomic potential. The SOC term on the same atom is taken into account and it can be obtained by calculating the mean value:

3 $${H}_{i,\alpha \beta }^{SO}={\lambda }_{i}{\langle {\bf{L}}\cdot \sigma \rangle }_{\alpha \beta },$$

where *λ*_*i*_ is the SOC strength of the *i*-th atom and **L** is the orbital angular momentum operator. The matrix element ${\langle {\bf{L}}\cdot \sigma \rangle }_{\alpha \beta }$ is given in the basis of atomic orbitals (*α*, *β*), and the dimensionless SOC operator **L** ⋅ *σ* for the relevant orbitals (4*s*, 4*p*_*x*_, and 4*p*_*y*_) in the 2D system is given by

4 $${\bf{L}}\cdot \sigma =(\begin{array}{ccc}0 & 0 & 0\\ 0 & 0 & -i{s}_{z}\\ 0 & i{s}_{z} & 0\end{array}),$$

where $${s}_{z}=(\begin{array}{cc}1 & 0\\ 0 & -\,1\end{array})$$. The SOC strengths of Ga and As atoms are chosen to be 0.058 eV and 0.140 eV, respectively^86^.

### Peierls substitution and a composite electric and magnetic fields

When a uniform perpendicular magnetic field, ${\bf{B}}={B}_{z}\hat{z}$, is applied to monolayer GaAs, the effective Hamiltonian could be regarded as the Peierls substitution Hamiltonian^87^. Each Hamiltonian matrix element turns into the product of the zero-field element and the extra Peierls phase, exp(*i*2*πθ*_*mn*_), where ${\theta }_{mn}=\mathrm{(1}/{\varphi }_{0})\,{\int }_{m}^{n}\,{\bf{A}}\cdot d{\bf{l}}$ is a line integral of the vector potential **A** from the *m*-th to *n*-th site, **A** is chosen as (0, *B*_*z*_*x*, 0) in the Landau gauge, and *ϕ*_0_ = *h*/*e* (4.1357 × 10^−15^ T · m^2^) is the magnetic flux quantum. The unit cell becomes an enlarged rectangle with 2*R*_*B*_ Ga and 2*R*_*B*_ As atoms to satisfy the periodicity of Peierls phase, where ${R}_{B}={\varphi }_{0}/\varphi ={\varphi }_{0}/({B}_{z}\sqrt{3}/2{a}^{2})\sim 26739\,T/{B}_{z}$ is the ratio of flux quantum to magnetic flux through a hexagon *ϕ* [Fig. 2(a)]. The reduced Brillouin zone has an area of $4{\pi }^{2}/\sqrt{3}{a}^{2}{R}_{B}$. The magnetic Hamiltonian is built in the space spanned by the 24*R*_*B*_ tight-binding functions $\{|G{a}_{m}^{orb}\rangle ,|A{s}_{m}^{orb}\rangle$; $m=1,2,3,\ldots ,2{R}_{B}$; $orb=4s,4{p}_{x},4{p}_{y}\}\otimes \{\uparrow ,\downarrow \}$. An electric field ${\bf{E}}={E}_{z}\hat{z}$ along the *z*-axis introduces a Coulomb potential energy −*eE*_*z*_*l*_*z*_/2 (*eE*_*z*_*l*_*z*_/2) to the site energy of the Ga (As) sublattice. The exact diagonalization method of the giant Hamiltonian matrix ${\mathcal H}$ yields the energy spectrum *E*^*c*,*v*^ and wave functions |Ψ^*c*,*v*^〉, where the superscripts *c* and *v* denote the conduction and valence subbands, respectively.
